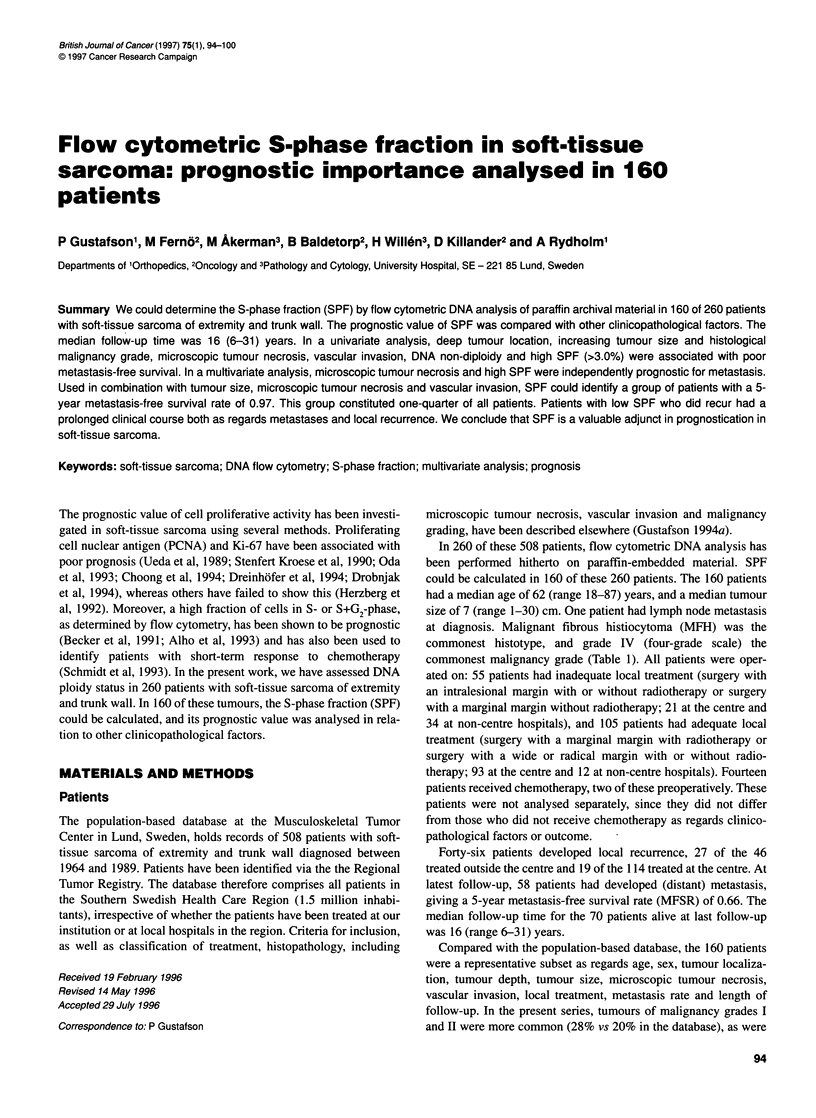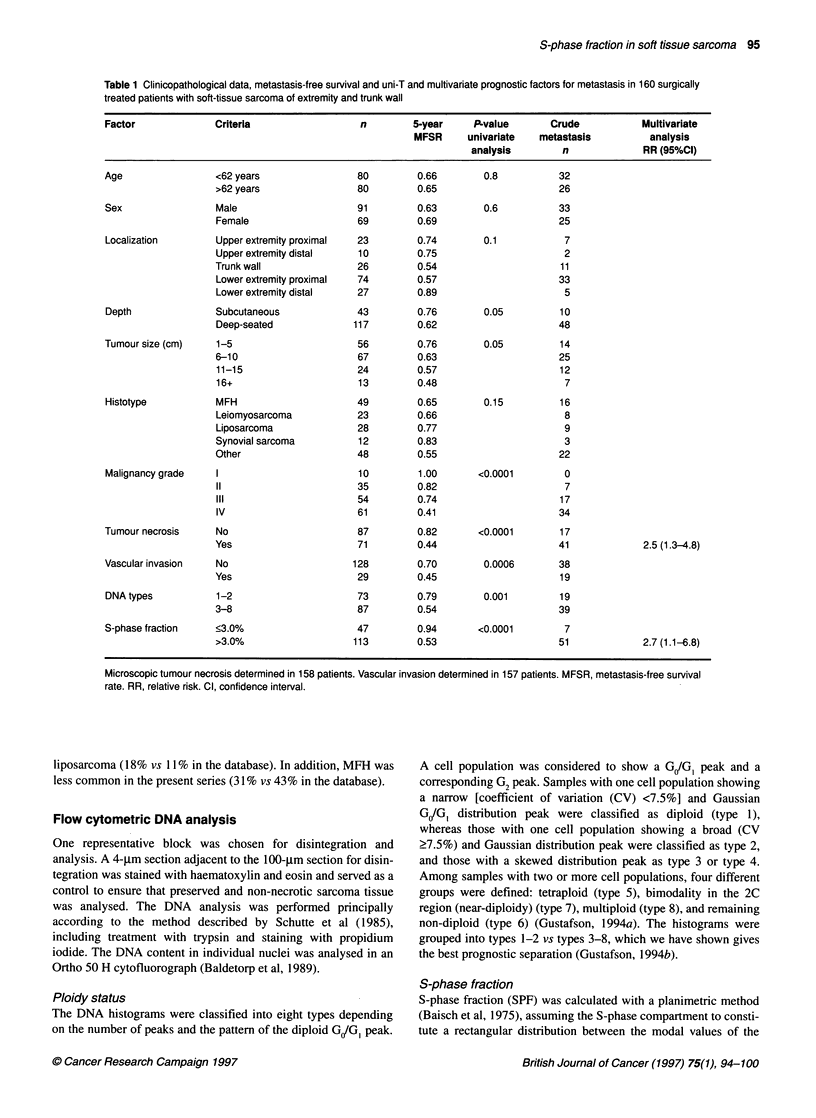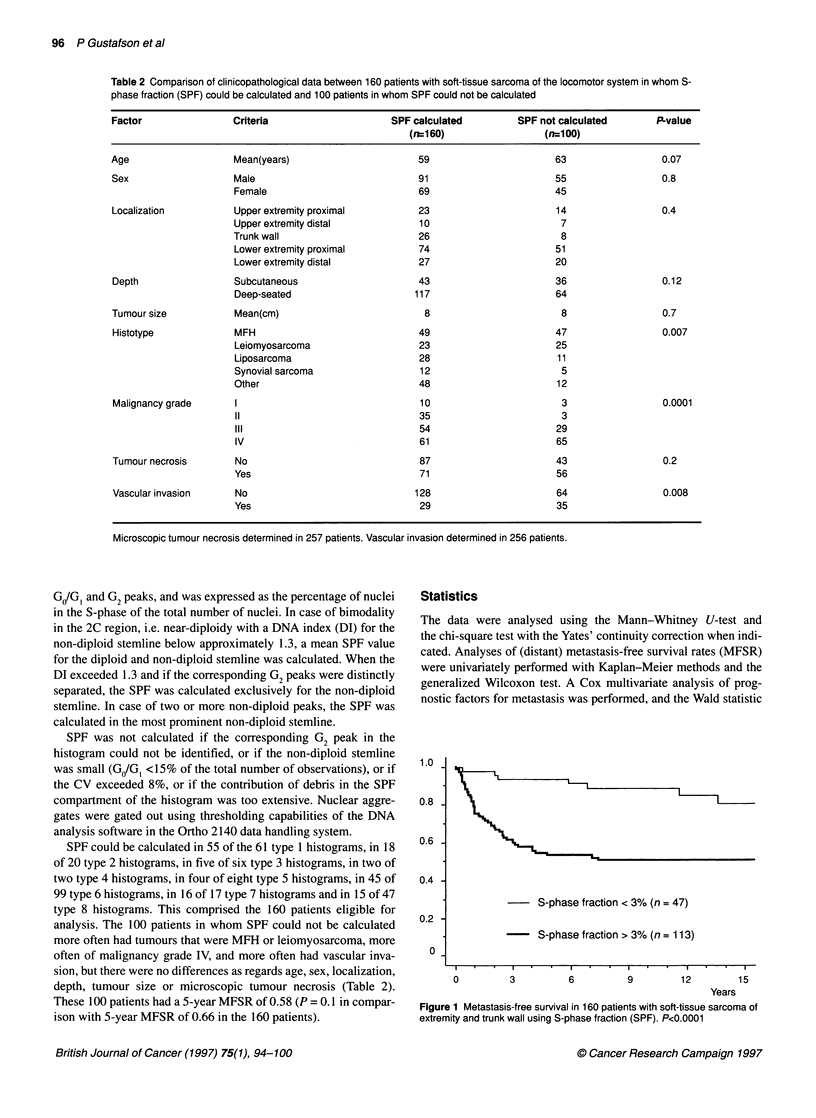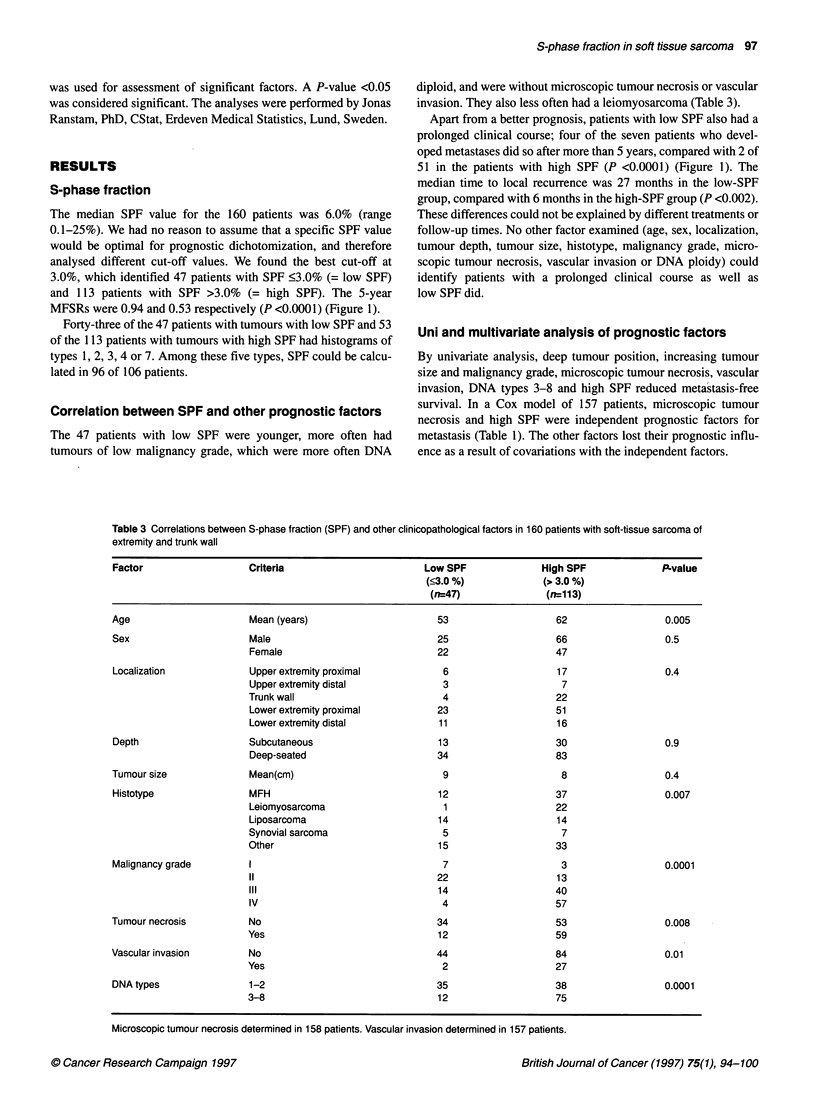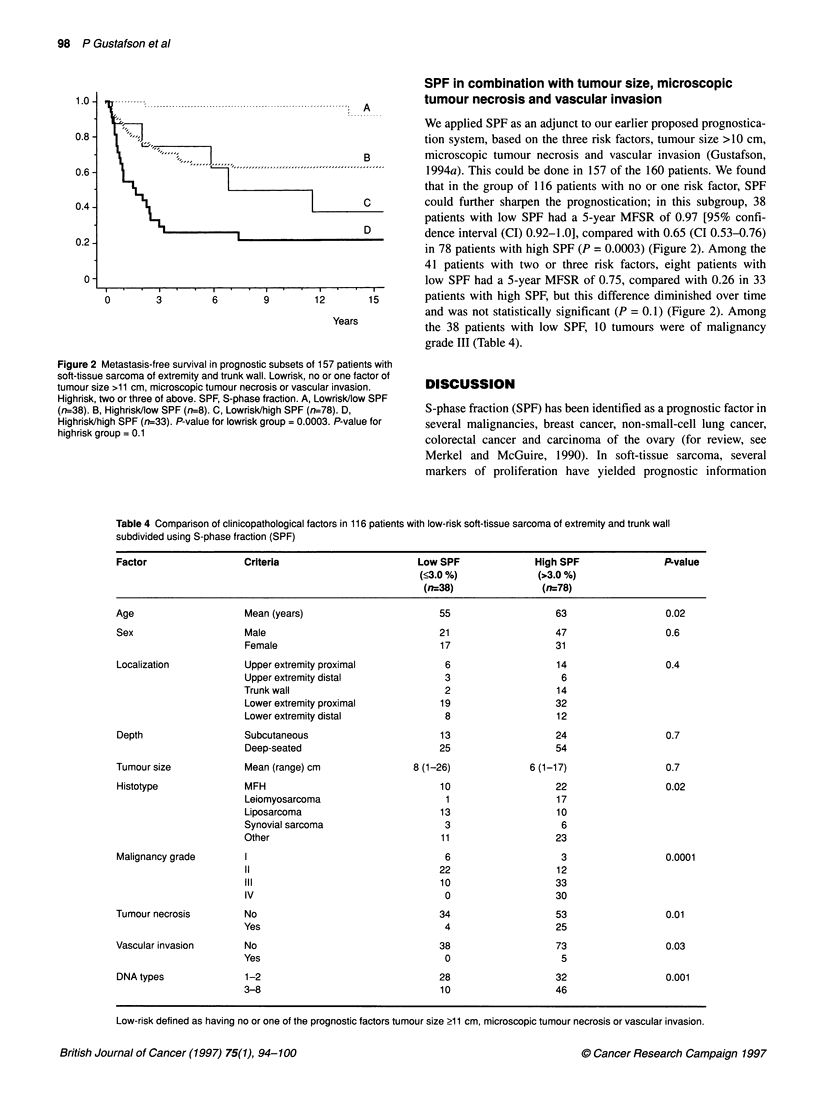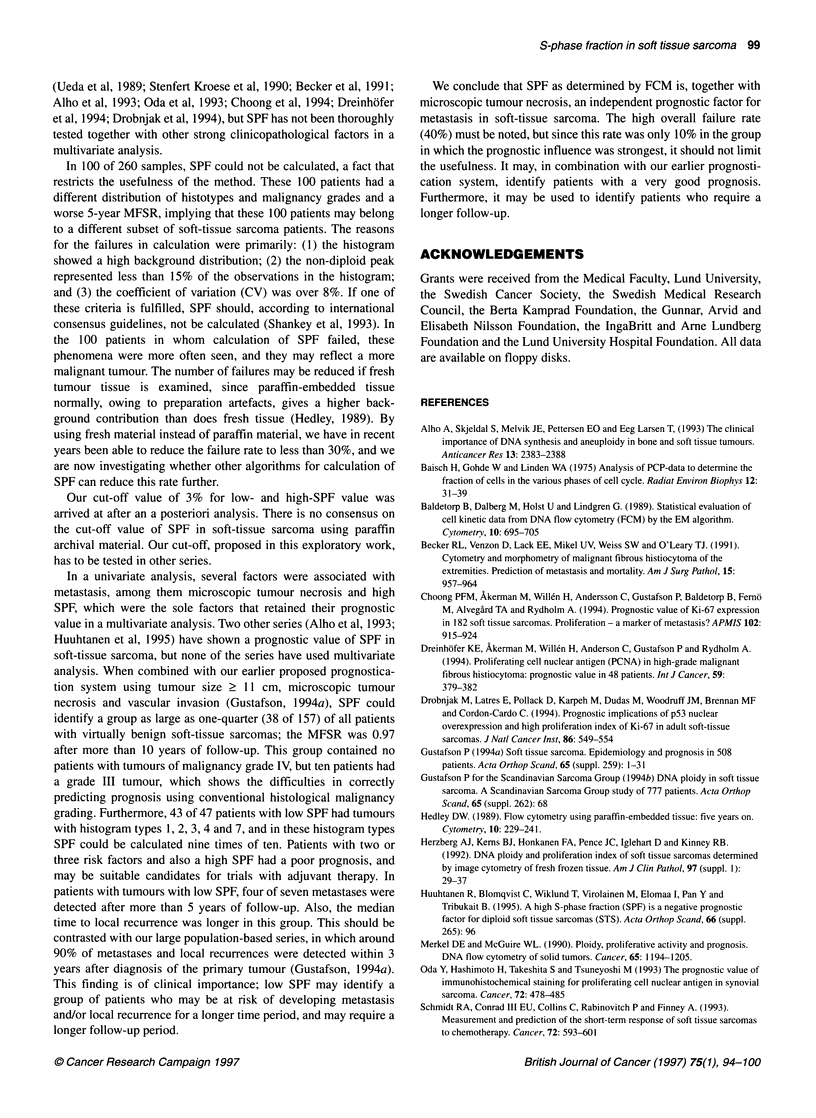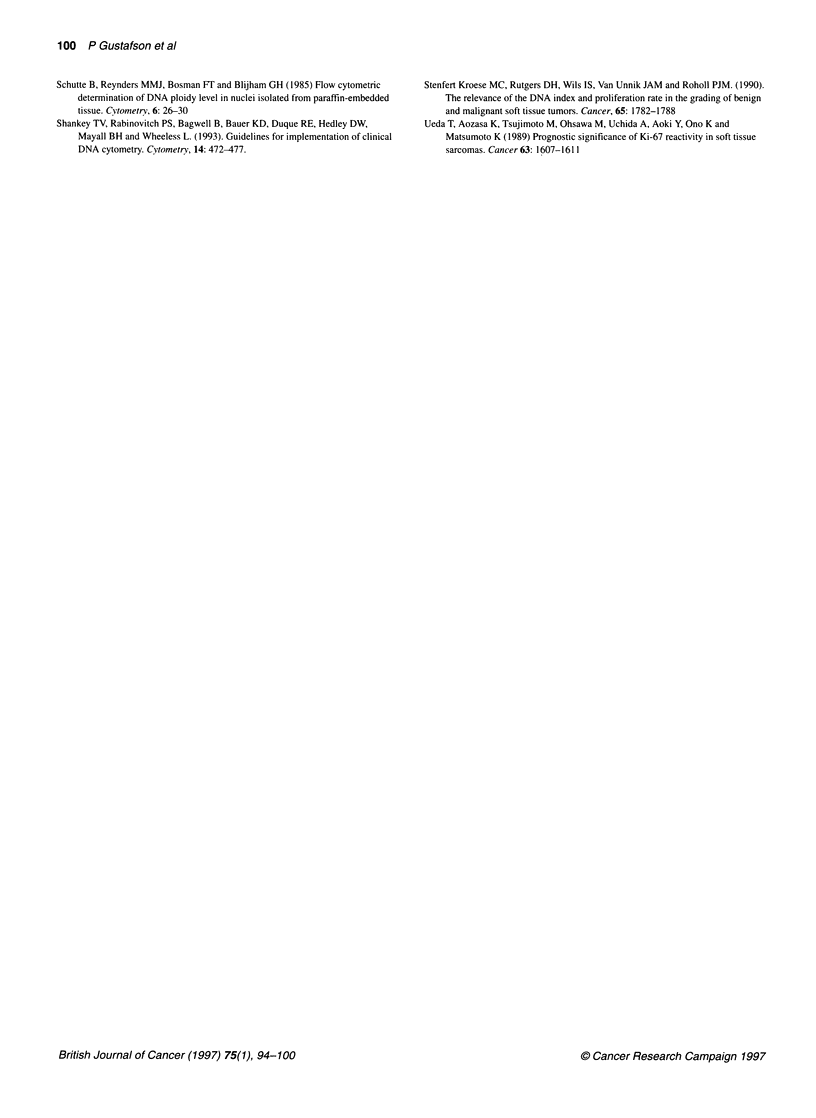# Flow cytometric S-phase fraction in soft-tissue sarcoma: prognostic importance analysed in 160 patients.

**DOI:** 10.1038/bjc.1997.15

**Published:** 1997

**Authors:** P. Gustafson, M. FernÃ¶, M. Akerman, B. Baldetorp, H. WillÃ©n, D. Killander, A. Rydholm

**Affiliations:** Department of Orthopedics, University Hospital, Lund, Sweden.

## Abstract

We could determine the S-phase fraction (SPF) by flow cytometric DNA analysis of paraffin archival material in 160 of 260 patients with soft-tissue sarcoma of extremity and trunk wall. The prognostic value of SPF was compared with other clinicopathological factors. The median follow-up time was 16 (6-31) years. In a univariate analysis, deep tumour location, increasing tumour size and histological malignancy grade, microscopic tumour necrosis, vascular invasion, DNA non-diploidy and high SPF (>3.0%) were associated with poor metastasis-free survival. In a multivariate analysis, microscopic tumour necrosis and high SPF were independently prognostic for metastasis. Used in combination with tumour size, microscopic tumour necrosis and vascular invasion, SPF could identify a group of patients with a 5-year metastasis-free survival rate of 0.97. This group constituted one-quarter of all patients. Patients with low SPF who did recur had a prolonged clinical course both as regards metastases and local recurrence. We conclude that SPF is a valuable adjunct in prognostication in soft-tissue sarcoma.


British Journal of Cancer (1997) 75(1), 94–100
© 1997 Cancer Research Campaign

### Flow cytometric S-phase fraction in soft-tissue sarcoma: prognostic importance analysed in 160 patients

P Gustafson[1], M Fernö[2], M Åkerman[3], B Baldetorp[2], H Willén[3], D Killander[2] and A Rydholm[1]

Departments of [1]Orthopedics, [2]Oncology and [3]Pathology and Cytology, University Hospital, SE – 221 85 Lund, Sweden

**Summary** We could determine the S-phase fraction (SPF) by flow cytometric DNA analysis of paraffin archival material in 160 of 260 patients with soft-tissue sarcoma of extremity and trunk wall. The prognostic value of SPF was compared with other clinicopathological factors. The median follow-up time was 16 (6–31) years. In a univariate analysis, deep tumour location, increasing tumour size and histological malignancy grade, microscopic tumour necrosis, vascular invasion, DNA non-diploidy and high SPF (>3.0%) were associated with poor metastasis-free survival. In a multivariate analysis, microscopic tumour necrosis and high SPF were independently prognostic for metastasis. Used in combination with tumour size, microscopic tumour necrosis and vascular invasion, SPF could identify a group of patients with a 5-year metastasis-free survival rate of 0.97. This group constituted one-quarter of all patients. Patients with low SPF who did recur had a prolonged clinical course both as regards metastases and local recurrence. We conclude that SPF is a valuable adjunct in prognostication in soft-tissue sarcoma.

**Keywords:** soft-tissue sarcoma; DNA flow cytometry; S-phase fraction; multivariate analysis; prognosis

The prognostic value of cell proliferative activity has been investigated in soft-tissue sarcoma using several methods. Proliferating cell nuclear antigen (PCNA) and Ki-67 have been associated with poor prognosis (Ueda et al, 1989; Stenfert Kroese et al, 1990; Oda et al, 1993; Choong et al, 1994; Dreinhöfer et al, 1994; Drobnjak et al, 1994), whereas others have failed to show this (Herzberg et al, 1992). Moreover, a high fraction of cells in S- or S+$G_2$-phase, as determined by flow cytometry, has been shown to be prognostic (Becker et al, 1991; Alho et al, 1993) and has also been used to identify patients with short-term response to chemotherapy (Schmidt et al, 1993). In the present work, we have assessed DNA ploidy status in 260 patients with soft-tissue sarcoma of extremity and trunk wall. In 160 of these tumours, the S-phase fraction (SPF) could be calculated, and its prognostic value was analysed in relation to other clinicopathological factors.

### MATERIALS AND METHODS

### Patients

The population-based database at the Musculoskeletal Tumor Center in Lund, Sweden, holds records of 508 patients with soft-tissue sarcoma of extremity and trunk wall diagnosed between 1964 and 1989. Patients have been identified via the the Regional Tumor Registry. The database therefore comprises all patients in the Southern Swedish Health Care Region (1.5 million inhabitants), irrespective of whether the patients have been treated at our institution or at local hospitals in the region. Criteria for inclusion, as well as classification of treatment, histopathology, including microscopic tumour necrosis, vascular invasion and malignancy grading, have been described elsewhere (Gustafson 1994*a*).

In 260 of these 508 patients, flow cytometric DNA analysis has been performed hitherto on paraffin-embedded material. SPF could be calculated in 160 of these 260 patients. The 160 patients had a median age of 62 (range 18–87) years, and a median tumour size of 7 (range 1–30) cm. One patient had lymph node metastasis at diagnosis. Malignant fibrous histiocytoma (MFH) was the commonest histotype, and grade IV (four-grade scale) the commonest malignancy grade (Table 1). All patients were operated on: 55 patients had inadequate local treatment (surgery with an intralesional margin with or without radiotherapy or surgery with a marginal margin without radiotherapy; 21 at the centre and 34 at non-centre hospitals), and 105 patients had adequate local treatment (surgery with a marginal margin with radiotherapy or surgery with a wide or radical margin with or without radiotherapy; 93 at the centre and 12 at non-centre hospitals). Fourteen patients received chemotherapy, two of these preoperatively. These patients were not analysed separately, since they did not differ from those who did not receive chemotherapy as regards clinicopathological factors or outcome.

Forty-six patients developed local recurrence, 27 of the 46 treated outside the centre and 19 of the 114 treated at the centre. At latest follow-up, 58 patients had developed (distant) metastasis, giving a 5-year metastasis-free survival rate (MFSR) of 0.66. The median follow-up time for the 70 patients alive at last follow-up was 16 (range 6–31) years.

Compared with the population-based database, the 160 patients were a representative subset as regards age, sex, tumour localization, tumour depth, tumour size, microscopic tumour necrosis, vascular invasion, local treatment, metastasis rate and length of follow-up. In the present series, tumours of malignancy grades I and II were more common (28% *vs* 20% in the database), as were

*Received 19 February 1996*
*Revised 14 May 1996*
*Accepted 29 July 1996*

*Correspondence to:* P Gustafson

**Table 1** Clinicopathological data, metastasis-free survival and uni-T and multivariate prognostic factors for metastasis in 160 surgically treated patients with soft-tissue sarcoma of extremity and trunk wall

| Factor | Criteria | *n* | 5-year MFSR | *P*-value univariate analysis | Crude metastasis *n* | Multivariate analysis RR (95%CI) |
|---|---|---|---|---|---|---|
| Age | <62 years | 80 | 0.66 | 0.8 | 32 | |
| | >62 years | 80 | 0.65 | | 26 | |
| Sex | Male | 91 | 0.63 | 0.6 | 33 | |
| | Female | 69 | 0.69 | | 25 | |
| Localization | Upper extremity proximal | 23 | 0.74 | 0.1 | 7 | |
| | Upper extremity distal | 10 | 0.75 | | 2 | |
| | Trunk wall | 26 | 0.54 | | 11 | |
| | Lower extremity proximal | 74 | 0.57 | | 33 | |
| | Lower extremity distal | 27 | 0.89 | | 5 | |
| Depth | Subcutaneous | 43 | 0.76 | 0.05 | 10 | |
| | Deep-seated | 117 | 0.62 | | 48 | |
| Tumour size (cm) | 1–5 | 56 | 0.76 | 0.05 | 14 | |
| | 6–10 | 67 | 0.63 | | 25 | |
| | 11–15 | 24 | 0.57 | | 12 | |
| | 16+ | 13 | 0.48 | | 7 | |
| Histotype | MFH | 49 | 0.65 | 0.15 | 16 | |
| | Leiomyosarcoma | 23 | 0.66 | | 8 | |
| | Liposarcoma | 28 | 0.77 | | 9 | |
| | Synovial sarcoma | 12 | 0.83 | | 3 | |
| | Other | 48 | 0.55 | | 22 | |
| Malignancy grade | I | 10 | 1.00 | <0.0001 | 0 | |
| | II | 35 | 0.82 | | 7 | |
| | III | 54 | 0.74 | | 17 | |
| | IV | 61 | 0.41 | | 34 | |
| Tumour necrosis | No | 87 | 0.82 | <0.0001 | 17 | |
| | Yes | 71 | 0.44 | | 41 | 2.5 (1.3–4.8) |
| Vascular invasion | No | 128 | 0.70 | 0.0006 | 38 | |
| | Yes | 29 | 0.45 | | 19 | |
| DNA types | 1–2 | 73 | 0.79 | 0.001 | 19 | |
| | 3–8 | 87 | 0.54 | | 39 | |
| S-phase fraction | ≤3.0% | 47 | 0.94 | <0.0001 | 7 | |
| | >3.0% | 113 | 0.53 | | 51 | 2.7 (1.1–6.8) |

Microscopic tumour necrosis determined in 158 patients. Vascular invasion determined in 157 patients. MFSR, metastasis-free survival rate. RR, relative risk. CI, confidence interval.

liposarcoma (18% *vs* 11% in the database). In addition, MFH was less common in the present series (31% *vs* 43% in the database).

### Flow cytometric DNA analysis

One representative block was chosen for disintegration and analysis. A 4-μm section adjacent to the 100-μm section for disintegration was stained with haematoxylin and eosin and served as a control to ensure that preserved and non-necrotic sarcoma tissue was analysed. The DNA analysis was performed principally according to the method described by Schutte et al (1985), including treatment with trypsin and staining with propidium iodide. The DNA content in individual nuclei was analysed in an Ortho 50 H cytofluorograph (Baldetorp et al, 1989).

### *Ploidy status*

The DNA histograms were classified into eight types depending on the number of peaks and the pattern of the diploid $G_0/G_1$ peak. A cell population was considered to show a $G_0/G_1$ peak and a corresponding $G_2$ peak. Samples with one cell population showing a narrow [coefficient of variation (CV) <7.5%] and Gaussian $G_0/G_1$ distribution peak were classified as diploid (type 1), whereas those with one cell population showing a broad (CV ≥7.5%) and Gaussian distribution peak were classified as type 2, and those with a skewed distribution peak as type 3 or type 4. Among samples with two or more cell populations, four different groups were defined: tetraploid (type 5), bimodality in the 2C region (near-diploidy) (type 7), multiploid (type 8), and remaining non-diploid (type 6) (Gustafson, 1994*a*). The histograms were grouped into types 1–2 *vs* types 3–8, which we have shown gives the best prognostic separation (Gustafson, 1994*b*).

### *S-phase fraction*

S-phase fraction (SPF) was calculated with a planimetric method (Baisch et al, 1975), assuming the S-phase compartment to constitute a rectangular distribution between the modal values of the

© Cancer Research Campaign 1997

**Table 2** Comparison of clinicopathological data between 160 patients with soft-tissue sarcoma of the locomotor system in whom S-phase fraction (SPF) could be calculated and 100 patients in whom SPF could not be calculated

| Factor | Criteria | SPF calculated ($n$=160) | SPF not calculated ($n$=100) | $P$-value |
|---|---|---|---|---|
| Age | Mean(years) | 59 | 63 | 0.07 |
| Sex | Male | 91 | 55 | 0.8 |
| | Female | 69 | 45 | |
| Localization | Upper extremity proximal | 23 | 14 | 0.4 |
| | Upper extremity distal | 10 | 7 | |
| | Trunk wall | 26 | 8 | |
| | Lower extremity proximal | 74 | 51 | |
| | Lower extremity distal | 27 | 20 | |
| Depth | Subcutaneous | 43 | 36 | 0.12 |
| | Deep-seated | 117 | 64 | |
| Tumour size | Mean(cm) | 8 | 8 | 0.7 |
| Histotype | MFH | 49 | 47 | 0.007 |
| | Leiomyosarcoma | 23 | 25 | |
| | Liposarcoma | 28 | 11 | |
| | Synovial sarcoma | 12 | 5 | |
| | Other | 48 | 12 | |
| Malignancy grade | I | 10 | 3 | 0.0001 |
| | II | 35 | 3 | |
| | III | 54 | 29 | |
| | IV | 61 | 65 | |
| Tumour necrosis | No | 87 | 43 | 0.2 |
| | Yes | 71 | 56 | |
| Vascular invasion | No | 128 | 64 | 0.008 |
| | Yes | 29 | 35 | |

Microscopic tumour necrosis determined in 257 patients. Vascular invasion determined in 256 patients.

$G_0/G_1$ and $G_2$ peaks, and was expressed as the percentage of nuclei in the S-phase of the total number of nuclei. In case of bimodality in the 2C region, i.e. near-diploidy with a DNA index (DI) for the non-diploid stemline below approximately 1.3, a mean SPF value for the diploid and non-diploid stemline was calculated. When the DI exceeded 1.3 and if the corresponding $G_2$ peaks were distinctly separated, the SPF was calculated exclusively for the non-diploid stemline. In case of two or more non-diploid peaks, the SPF was calculated in the most prominent non-diploid stemline.

SPF was not calculated if the corresponding $G_2$ peak in the histogram could not be identified, or if the non-diploid stemline was small ($G_0/G_1$ <15% of the total number of observations), or if the CV exceeded 8%, or if the contribution of debris in the SPF compartment of the histogram was too extensive. Nuclear aggregates were gated out using thresholding capabilities of the DNA analysis software in the Ortho 2140 data handling system.

SPF could be calculated in 55 of the 61 type 1 histograms, in 18 of 20 type 2 histograms, in five of six type 3 histograms, in two of two type 4 histograms, in four of eight type 5 histograms, in 45 of 99 type 6 histograms, in 16 of 17 type 7 histograms and in 15 of 47 type 8 histograms. This comprised the 160 patients eligible for analysis. The 100 patients in whom SPF could not be calculated more often had tumours that were MFH or leiomyosarcoma, more often of malignancy grade IV, and more often had vascular invasion, but there were no differences as regards age, sex, localization, depth, tumour size or microscopic tumour necrosis (Table 2). These 100 patients had a 5-year MFSR of 0.58 ($P$ = 0.1 in comparison with 5-year MFSR of 0.66 in the 160 patients).

### Statistics

The data were analysed using the Mann–Whitney $U$-test and the chi-square test with the Yates' continuity correction when indicated. Analyses of (distant) metastasis-free survival rates (MFSR) were univariately performed with Kaplan–Meier methods and the generalized Wilcoxon test. A Cox multivariate analysis of prognostic factors for metastasis was performed, and the Wald statistic

**Figure 1** Metastasis-free survival in 160 patients with soft-tissue sarcoma of extremity and trunk wall using S-phase fraction (SPF). $P$<0.0001

© Cancer Research Campaign 1997

was used for assessment of significant factors. A $P$-value <0.05 was considered significant. The analyses were performed by Jonas Ranstam, PhD, CStat, Erdeven Medical Statistics, Lund, Sweden.

### RESULTS

### S-phase fraction

The median SPF value for the 160 patients was 6.0% (range 0.1–25%). We had no reason to assume that a specific SPF value would be optimal for prognostic dichotomization, and therefore analysed different cut-off values. We found the best cut-off at 3.0%, which identified 47 patients with SPF ≤3.0% (= low SPF) and 113 patients with SPF >3.0% (= high SPF). The 5-year MFSRs were 0.94 and 0.53 respectively ($P$ <0.0001) (Figure 1).

Forty-three of the 47 patients with tumours with low SPF and 53 of the 113 patients with tumours with high SPF had histograms of types 1, 2, 3, 4 or 7. Among these five types, SPF could be calculated in 96 of 106 patients.

### Correlation between SPF and other prognostic factors

The 47 patients with low SPF were younger, more often had tumours of low malignancy grade, which were more often DNA diploid, and were without microscopic tumour necrosis or vascular invasion. They also less often had a leiomyosarcoma (Table 3).

Apart from a better prognosis, patients with low SPF also had a prolonged clinical course; four of the seven patients who developed metastases did so after more than 5 years, compared with 2 of 51 in the patients with high SPF ($P$ <0.0001) (Figure 1). The median time to local recurrence was 27 months in the low-SPF group, compared with 6 months in the high-SPF group ($P$ <0.002). These differences could not be explained by different treatments or follow-up times. No other factor examined (age, sex, localization, tumour depth, tumour size, histotype, malignancy grade, microscopic tumour necrosis, vascular invasion or DNA ploidy) could identify patients with a prolonged clinical course as well as low SPF did.

### Uni and multivariate analysis of prognostic factors

By univariate analysis, deep tumour position, increasing tumour size and malignancy grade, microscopic tumour necrosis, vascular invasion, DNA types 3–8 and high SPF reduced metastasis-free survival. In a Cox model of 157 patients, microscopic tumour necrosis and high SPF were independent prognostic factors for metastasis (Table 1). The other factors lost their prognostic influence as a result of covariations with the independent factors.

**Table 3** Correlations between S-phase fraction (SPF) and other clinicopathological factors in 160 patients with soft-tissue sarcoma of extremity and trunk wall

| Factor | Criteria | Low SPF (≤3.0 %) ($n$=47) | High SPF (> 3.0 %) ($n$=113) | $P$-value |
|---|---|---|---|---|
| Age | Mean (years) | 53 | 62 | 0.005 |
| Sex | Male | 25 | 66 | 0.5 |
| | Female | 22 | 47 | |
| Localization | Upper extremity proximal | 6 | 17 | 0.4 |
| | Upper extremity distal | 3 | 7 | |
| | Trunk wall | 4 | 22 | |
| | Lower extremity proximal | 23 | 51 | |
| | Lower extremity distal | 11 | 16 | |
| Depth | Subcutaneous | 13 | 30 | 0.9 |
| | Deep-seated | 34 | 83 | |
| Tumour size | Mean(cm) | 9 | 8 | 0.4 |
| Histotype | MFH | 12 | 37 | 0.007 |
| | Leiomyosarcoma | 1 | 22 | |
| | Liposarcoma | 14 | 14 | |
| | Synovial sarcoma | 5 | 7 | |
| | Other | 15 | 33 | |
| Malignancy grade | I | 7 | 3 | 0.0001 |
| | II | 22 | 13 | |
| | III | 14 | 40 | |
| | IV | 4 | 57 | |
| Tumour necrosis | No | 34 | 53 | 0.008 |
| | Yes | 12 | 59 | |
| Vascular invasion | No | 44 | 84 | 0.01 |
| | Yes | 2 | 27 | |
| DNA types | 1–2 | 35 | 38 | 0.0001 |
| | 3–8 | 12 | 75 | |

Microscopic tumour necrosis determined in 158 patients. Vascular invasion determined in 157 patients.

© Cancer Research Campaign 1997

**Figure 2** Metastasis-free survival in prognostic subsets of 157 patients with soft-tissue sarcoma of extremity and trunk wall. Lowrisk, no or one factor of tumour size >11 cm, microscopic tumour necrosis or vascular invasion. Highrisk, two or three of above. SPF, S-phase fraction. A, Lowrisk/low SPF (*n*=38). B, Highrisk/low SPF (*n*=8). C, Lowrisk/high SPF (*n*=78). D, Highrisk/high SPF (*n*=33). *P*-value for lowrisk group = 0.0003. *P*-value for highrisk group = 0.1

### SPF in combination with tumour size, microscopic tumour necrosis and vascular invasion

We applied SPF as an adjunct to our earlier proposed prognostication system, based on the three risk factors, tumour size >10 cm, microscopic tumour necrosis and vascular invasion (Gustafson, 1994*a*). This could be done in 157 of the 160 patients. We found that in the group of 116 patients with no or one risk factor, SPF could further sharpen the prognostication; in this subgroup, 38 patients with low SPF had a 5-year MFSR of 0.97 [95% confidence interval (CI) 0.92–1.0], compared with 0.65 (CI 0.53–0.76) in 78 patients with high SPF ($P$ = 0.0003) (Figure 2). Among the 41 patients with two or three risk factors, eight patients with low SPF had a 5-year MFSR of 0.75, compared with 0.26 in 33 patients with high SPF, but this difference diminished over time and was not statistically significant ($P$ = 0.1) (Figure 2). Among the 38 patients with low SPF, 10 tumours were of malignancy grade III (Table 4).

### DISCUSSION

S-phase fraction (SPF) has been identified as a prognostic factor in several malignancies, breast cancer, non-small-cell lung cancer, colorectal cancer and carcinoma of the ovary (for review, see Merkel and McGuire, 1990). In soft-tissue sarcoma, several markers of proliferation have yielded prognostic information

**Table 4** Comparison of clinicopathological factors in 116 patients with low-risk soft-tissue sarcoma of extremity and trunk wall subdivided using S-phase fraction (SPF)

| Factor | Criteria | Low SPF (≤3.0 %) (*n*=38) | High SPF (>3.0 %) (*n*=78) | *P*-value |
|---|---|---|---|---|
| Age | Mean (years) | 55 | 63 | 0.02 |
| Sex | Male | 21 | 47 | 0.6 |
| | Female | 17 | 31 | |
| Localization | Upper extremity proximal | 6 | 14 | 0.4 |
| | Upper extremity distal | 3 | 6 | |
| | Trunk wall | 2 | 14 | |
| | Lower extremity proximal | 19 | 32 | |
| | Lower extremity distal | 8 | 12 | |
| Depth | Subcutaneous | 13 | 24 | 0.7 |
| | Deep-seated | 25 | 54 | |
| Tumour size | Mean (range) cm | 8 (1–26) | 6 (1–17) | 0.7 |
| Histotype | MFH | 10 | 22 | 0.02 |
| | Leiomyosarcoma | 1 | 17 | |
| | Liposarcoma | 13 | 10 | |
| | Synovial sarcoma | 3 | 6 | |
| | Other | 11 | 23 | |
| Malignancy grade | I | 6 | 3 | 0.0001 |
| | II | 22 | 12 | |
| | III | 10 | 33 | |
| | IV | 0 | 30 | |
| Tumour necrosis | No | 34 | 53 | 0.01 |
| | Yes | 4 | 25 | |
| Vascular invasion | No | 38 | 73 | 0.03 |
| | Yes | 0 | 5 | |
| DNA types | 1–2 | 28 | 32 | 0.001 |
| | 3–8 | 10 | 46 | |

Low-risk defined as having no or one of the prognostic factors tumour size ≥11 cm, microscopic tumour necrosis or vascular invasion.

© Cancer Research Campaign 1997

(Ueda et al, 1989; Stenfert Kroese et al, 1990; Becker et al, 1991; Alho et al, 1993; Oda et al, 1993; Choong et al, 1994; Dreinhöfer et al, 1994; Drobnjak et al, 1994), but SPF has not been thoroughly tested together with other strong clinicopathological factors in a multivariate analysis.

In 100 of 260 samples, SPF could not be calculated, a fact that restricts the usefulness of the method. These 100 patients had a different distribution of histotypes and malignancy grades and a worse 5-year MFSR, implying that these 100 patients may belong to a different subset of soft-tissue sarcoma patients. The reasons for the failures in calculation were primarily: (1) the histogram showed a high background distribution; (2) the non-diploid peak represented less than 15% of the observations in the histogram; and (3) the coefficient of variation (CV) was over 8%. If one of these criteria is fulfilled, SPF should, according to international consensus guidelines, not be calculated (Shankey et al, 1993). In the 100 patients in whom calculation of SPF failed, these phenomena were more often seen, and they may reflect a more malignant tumour. The number of failures may be reduced if fresh tumour tissue is examined, since paraffin-embedded tissue normally, owing to preparation artefacts, gives a higher background contribution than does fresh tissue (Hedley, 1989). By using fresh material instead of paraffin material, we have in recent years been able to reduce the failure rate to less than 30%, and we are now investigating whether other algorithms for calculation of SPF can reduce this rate further.

Our cut-off value of 3% for low- and high-SPF value was arrived at after an a posteriori analysis. There is no consensus on the cut-off value of SPF in soft-tissue sarcoma using paraffin archival material. Our cut-off, proposed in this exploratory work, has to be tested in other series.

In a univariate analysis, several factors were associated with metastasis, among them microscopic tumour necrosis and high SPF, which were the sole factors that retained their prognostic value in a multivariate analysis. Two other series (Alho et al, 1993; Huuhtanen et al, 1995) have shown a prognostic value of SPF in soft-tissue sarcoma, but none of the series have used multivariate analysis. When combined with our earlier proposed prognostication system using tumour size $\geq$ 11 cm, microscopic tumour necrosis and vascular invasion (Gustafson, 1994*a*), SPF could identify a group as large as one-quarter (38 of 157) of all patients with virtually benign soft-tissue sarcomas; the MFSR was 0.97 after more than 10 years of follow-up. This group contained no patients with tumours of malignancy grade IV, but ten patients had a grade III tumour, which shows the difficulties in correctly predicting prognosis using conventional histological malignancy grading. Furthermore, 43 of 47 patients with low SPF had tumours with histogram types 1, 2, 3, 4 and 7, and in these histogram types SPF could be calculated nine times of ten. Patients with two or three risk factors and also a high SPF had a poor prognosis, and may be suitable candidates for trials with adjuvant therapy. In patients with tumours with low SPF, four of seven metastases were detected after more than 5 years of follow-up. Also, the median time to local recurrence was longer in this group. This should be contrasted with our large population-based series, in which around 90% of metastases and local recurrences were detected within 3 years after diagnosis of the primary tumour (Gustafson, 1994*a*). This finding is of clinical importance; low SPF may identify a group of patients who may be at risk of developing metastasis and/or local recurrence for a longer time period, and may require a longer follow-up period.

We conclude that SPF as determined by FCM is, together with microscopic tumour necrosis, an independent prognostic factor for metastasis in soft-tissue sarcoma. The high overall failure rate (40%) must be noted, but since this rate was only 10% in the group in which the prognostic influence was strongest, it should not limit the usefulness. It may, in combination with our earlier prognostication system, identify patients with a very good prognosis. Furthermore, it may be used to identify patients who require a longer follow-up.

### ACKNOWLEDGEMENTS

Grants were received from the Medical Faculty, Lund University, the Swedish Cancer Society, the Swedish Medical Research Council, the Berta Kamprad Foundation, the Gunnar, Arvid and Elisabeth Nilsson Foundation, the IngaBritt and Arne Lundberg Foundation and the Lund University Hospital Foundation. All data are available on floppy disks.

### REFERENCES

Alho A, Skjeldal S, Melvik JE, Pettersen EO and Eeg Larsen T, (1993) The clinical importance of DNA synthesis and aneuploidy in bone and soft tissue tumours. *Anticancer Res* **13**: 2383–2388

Baisch H, Gohde W and Linden WA (1975) Analysis of PCP-data to determine the fraction of cells in the various phases of cell cycle. *Radiat Environ Biophys* **12**: 31–39

Baldetorp B, Dalberg M, Holst U and Lindgren G. (1989). Statistical evaluation of cell kinetic data from DNA flow cytometry (FCM) by the EM algorithm. *Cytometry*, **10**: 695–705

Becker RL, Venzon D, Lack EE, Mikel UV, Weiss SW and O'Leary TJ. (1991). Cytometry and morphometry of malignant fibrous histiocytoma of the extremities. Prediction of metastasis and mortality. *Am J Surg Pathol*, **15**: 957–964

Choong PFM, Åkerman M, Willén H, Andersson C, Gustafson P, Baldetorp B, Fernö M, Alvegård TA and Rydholm A. (1994). Prognostic value of Ki-67 expression in 182 soft tissue sarcomas. Proliferation – a marker of metastasis? *APMIS* **102**: 915–924

Dreinhöfer KE, Åkerman M, Willén H, Anderson C, Gustafson P and Rydholm A. (1994). Proliferating cell nuclear antigen (PCNA) in high-grade malignant fibrous histiocytoma: prognostic value in 48 patients. *Int J Cancer*, **59**: 379–382

Drobnjak M, Latres E, Pollack D, Karpeh M, Dudas M, Woodruff JM, Brennan MF and Cordon-Cardo C. (1994). Prognostic implications of p53 nuclear overexpression and high proliferation index of Ki-67 in adult soft-tissue sarcomas. *J Natl Cancer Inst*, **86**: 549–554

Gustafson P (1994*a*) Soft tissue sarcoma. Epidemiology and prognosis in 508 patients. *Acta Orthop Scand*, **65** (suppl. 259): 1–31

Gustafson P for the Scandinavian Sarcoma Group (1994*b*) DNA ploidy in soft tissue sarcoma. A Scandinavian Sarcoma Group study of 777 patients. *Acta Orthop Scand*, **65** (suppl. 262): 68

Hedley DW. (1989). Flow cytometry using paraffin-embedded tissue: five years on. *Cytometry*, **10**: 229–241.

Herzberg AJ, Kerns BJ, Honkanen FA, Pence JC, Iglehart D and Kinney RB. (1992). DNA ploidy and proliferation index of soft tissue sarcomas determined by image cytometry of fresh frozen tissue. *Am J Clin Pathol*, **97** (suppl. 1): 29–37

Huuhtanen R, Blomqvist C, Wiklund T, Virolainen M, Elomaa I, Pan Y and Tribukait B. (1995). A high S-phase fraction (SPF) is a negative prognostic factor for diploid soft tissue sarcomas (STS). *Acta Orthop Scand*, **66** (suppl. 265): 96

Merkel DE and McGuire WL. (1990). Ploidy, proliferative activity and prognosis. DNA flow cytometry of solid tumors. *Cancer*, **65**: 1194–1205.

Oda Y, Hashimoto H, Takeshita S and Tsuneyoshi M (1993) The prognostic value of immunohistochemical staining for proliferating cell nuclear antigen in synovial sarcoma. *Cancer*, **72**: 478–485

Schmidt RA, Conrad III EU, Collins C, Rabinovitch P and Finney A. (1993). Measurement and prediction of the short-term response of soft tissue sarcomas to chemotherapy. *Cancer*, **72**: 593–601

© Cancer Research Campaign 1997

Schutte B, Reynders MMJ, Bosman FT and Blijham GH (1985) Flow cytometric determination of DNA ploidy level in nuclei isolated from paraffin-embedded tissue. *Cytometry*, **6**: 26–30

Shankey TV, Rabinovitch PS, Bagwell B, Bauer KD, Duque RE, Hedley DW, Mayall BH and Wheeless L. (1993). Guidelines for implementation of clinical DNA cytometry. *Cytometry*, **14**: 472–477.

Stenfert Kroese MC, Rutgers DH, Wils IS, Van Unnik JAM and Roholl PJM. (1990). The relevance of the DNA index and proliferation rate in the grading of benign and malignant soft tissue tumors. *Cancer*, **65**: 1782–1788

Ueda T, Aozasa K, Tsujimoto M, Ohsawa M, Uchida A, Aoki Y, Ono K and Matsumoto K (1989) Prognostic significance of Ki-67 reactivity in soft tissue sarcomas. *Cancer* **63**: 1607–1611

*© Cancer Research Campaign 1997*